# Cork Oak Forests Soil Bacteria: Potential for Sustainable Agroforest Production

**DOI:** 10.3390/microorganisms9091973

**Published:** 2021-09-16

**Authors:** Francisca Reis, Ana João Pereira, Rui M. Tavares, Paula Baptista, Teresa Lino-Neto

**Affiliations:** 1 Plant Functional Biology Centre, BioSystems and Integrative Sciences Institute (BioISI), Campus de Gualtar, University of Minho, 4710-057 Braga, Portugal; franciscareis@bio.uminho.pt (F.R.); ana.pereira03@hotmail.com (A.J.P.); tavares@bio.uminho.pt (R.M.T.); 2 Centro de Investigação de Montanha (CIMO), Instituto Politécnico de Bragança, Campus de Santa Apolónia, 5300-253 Bragança, Portugal; pbaptista@ipb.pt

**Keywords:** plant growth promoting bacteria, cork oak, antagonism, biocontrol agent

## Abstract

Plant growth promoting rhizobacteria (PGPR) are in increasing demand due to their role in promoting sustainable practices, not only in agriculture but also in forestry. Keeping in mind the future application of PGPR for increasing cork oak sustainability, the aim of this study was to find cork oak PGPR isolates with increased nutrient solubilisation traits, able to promote root morphological changes and/or antagonize cork oak bark phytopathogens. Soils from three cork oak forests with distinct bioclimates (humid, semi-humid and semi-arid) were used for isolating bacteria. From the 7634 colony-forming units, 323 bacterial isolates were biochemically assayed for PGPR traits (siderophores production, phosphate solubilizing and organic acids production), and 51 were found to display all these traits. These PGPR were able to induce root morphological changes on *Arabidopsis thaliana*, like suppression of primary root growth, increase of lateral roots or root hairs formation. However, the most proficient PGPR displayed specific ability in changing a single root morphological trait. This ability was related not only to bacterial genotype, but also with the environment where bacteria thrived and isolation temperature. Bacteria from semi-arid environments (mainly *Bacillus megaterium* isolates) could hold a promising tool to enhance plant development. Other isolates (*Serratia quinivorens* or *B. cereus*) could be further explored for biocontrol purposes.

## 1. Introduction

Cork oak (*Quercus suber* L.) is a slow-growing and long-lived evergreen tree, native to western and central Mediterranean region, being mainly distributed along coastal regions of southwest Europe (France, Italy, Spain, and Portugal) and northwest Africa (Algeria, Morocco, and Tunisia; [1]). Cork oak forests cover almost 1.5 million ha in Europe, where Portugal has the largest distribution with nearly 740,000 ha and owning 50% of the global cork market [2]. Beyond their economic importance, these forests also host a remarkable biodiversity and are unique ecosystems recognized by their ecological value [3]. These woodlands are well adapted to the Mediterranean climate, where summers are warm and dry, and winters mild and wet. Over recent decades, a severe reduction in cork oak forests areas has been occurring, which is mainly due to the increasing of temperatures and long drought seasons [4], but also to changes in land use and new pathogen emergence [1]. Indeed, disease incidence and drought play the most significant role in trees health and forest ecosystem sustainability, where the consequences of water deficits further enhance fungal diseases [5]. An increase in cork oak diseases has been widely reported [6], including charcoal disease (caused by *Biscogniauxia mediterranea* (De Not.) Kuntze; Xylariales) and bot canker (caused by *Diplodia corticola* A.J.L. Phillips, A. Alves and J. Luque; Botryosphaeriales). Both *B. mediterranea* and *D. corticola* have been found in cork oak trees in a latent endophytic phase [7], only inducing disease symptoms when the plant host faces environmental stresses, like drought or high temperatures [5]. The spreading of these opportunistic pathogens is causing severe economic losses, but preventive measures for restricting cork oak charcoal disease and bot canker are still limited. The main currently adopted mechanism is the use of good phytosanitary practices [6].

With the upcoming increase in frequency and severity of drought events, new strategies for mitigating both abiotic and biotic stress effects on forests became crucial [4]. Beneficial microorganisms have been used as important partners for helping host plants to better tolerate these factors [8]. Among the sustainable developed strategies is the use of plant growth-promoting rhizobacteria (PGPR) that can benefit plant development by either direct or indirect mechanisms, including the reduction or prevention of harmful effects from phytopathogenic organisms [9]. Indeed, PGPR can act as biocontrol agents, protecting plants against phytopathogens by enhancing the defensive capacity of plants (triggering the induced systemic resistance responses) or by producing chemical compounds, such as hydrogen cyanide, antibiotics, bacteriocins and lytic enzymes [10]. Recent studies concerning bacterial communities in forest soils detected a regular presence of phyla, such as Proteobacteria, Actinobacteria, Firmicutes, and Acidobacteria [11,12,13]. Among Proteobacteria, rhizobacteria belonging to the genera *Azospirillum*, *Azotobacter*, *Burkholderia*, *Enterobacter*, *Klebsiella*, *Pseudomonas*, and *Serratia*, and those belonging to Actinobacteria genera (*Arthrobacter*, *Rhodococcus*, and *Streptomyces*), or even *Bacillus* from Firmicutes phylum, have all been reported to antagonize plant pathogens [14]. Besides contributing for the control of pathogens, rhizobacteria have been also described to directly facilitate plant growth and development [9]. Important direct growth promoting mechanisms include phosphate solubilisation, production of siderophores and organic acids [15]. Moreover, symbiotic soil bacteria have been recently described to be associated with cork oaks, contributing for their mycorrhization, and putatively enhancing ecosystem function [16].

Due to the multifunctional services provided by rhizobacteria, we investigated the functions of bacteria recovered from cork oak forest soils located in different landscapes and bioclimates. The idea was to capture the greatest possible diversity of bacteria that exhibit different ecological functions. We hypothesized that soil bacteria could provide benefits to cork oaks, either by enhancing their growth or by displaying biocontrol activities, thus contributing to their production and sustainability. Moreover, we anticipate that bacteria from semi-arid forests would be more tolerant to high temperatures. In this work, bacteria from three different cork oak forests in Portugal were isolated and biochemically characterized for PGPR traits. The most promising bacterial isolates were further evaluated for in-vivo plant growth promotion and antifungal activity against the cork oak bark pathogens, *B. mediterranea* and *D. corticola.* The selection of PGPR, well adapted to cork oak soils and displaying mechanisms for improving plant growth, would be a starting point for devising an optimized biofertilizer for enhancing soil fertility. Taking this into account, we based our selection on three of the most important mechanisms for improve agronomic features, such as pH regulation (production of organic acids), iron availability for plant absorption (siderophore production), and phosphate availability (second most important plant growth-limiting nutrient after nitrogen). To the best of our knowledge, this is the first report that combines biofertilizer and biocontrol traits for cork oak trees.

## 2. Material and Methods

### 2.1. Sample Collection and Bacterial Isolation

Sampling occurred during the autumn season (November and December 2019) in Portugal, at three different geographic locations—Grândola (GR), Limãos (LI), and Ermida (ER)—presenting three distinct bioclimates (semi-arid, sub-humid, and humid, respectively). Bioclimates were defined based on weather conditions, water availability levels, and local Emberger indexes (*Q*; [12,17]). Sampled cork oak forests presented sandy loam (GR and GR) or sandy clay loam (LI) soils with acidic properties (GR, pH 5.51; LI, pH 5.49; ER, pH 4.97). *Cistus* sp. was the main vegetation cover. Both GR and LI were agroforest systems where soil is yearly tilled, whereas ER was a wild forest with non-tilled soil. In each forest, three apparently healthy trees were selected at random, but at least 30 m apart from each other to avoid direct interlacing/connection of their roots. To collect soil samples, the uppermost layer of soil that consisted in plant litter and other organic material was removed. From each forest, three soil cores (5 cm in diameter and 10 cm in depth) were collected under the middle of cork oak canopy of three random trees, and all were thoroughly mixed together. At the end, three combined soil samples were obtained (including each, 3 cores × 3 trees samples), which were kept at 4 °C until processing.

In order to isolate PGPR, 1.0 g of each soil sample was transferred to a flask containing 10 mL of deionized water. After mixing, serial soil dilutions were prepared, and 100 µL aliquots (10^0^ to 10^−3^ dilutions) were spread onto Yeast extract Mannitol Agar selective solid medium (YMA), supplemented with 10 mL/L of aqueous solution of Congo-Red (CR; [18]). YMA-CR was used to grow PGPR colonies at 30 °C, 37 °C, and 45 °C, for 24 h and the number of Colony-Forming Units (CFU) were counted. Random bacterial isolates from each different soil sample and incubation temperature were further isolated on YMA-CR medium, using their respective growth temperature (30 °C, 37 °C or 45 °C), for 24 h. For increasing PGPR diversity, colonies displaying distinct morphological characteristics (form, colour, and elevation) were considered.

### 2.2. Plant Growth Promoting Biochemical Assays

Bacterial isolates were screened for siderophores and organic acids production, as well as for their phosphate solubilizing properties, using proper selective media. Bacteria capability to scavenge iron using siderophores was evaluated using Chrome Azurol Sulphonate (CAS) agar medium, which was prepared as described elsewhere [19]. Phosphate solubilizing microorganisms were screened using Pikovskaya (PVK) agar medium, supplemented with insoluble phosphate complexes (Ca_3_PO_4_ or AlPO_4_) and BromoPhenol Blue (BPB; [20,21]). To detect organic acids production, YMA agar medium supplemented with Bromothymol Blue (YMA-BB) was used [18]. After streaking isolates on these selective media, agar plates were incubated at 30 °C, for 96 h. Results were considered positive when a halo zone (colour change) on the selective media was noticeable around bacteria.

For detecting HCN (cyanogen) production, bacterial isolates that tested positive for all three previous biochemical tests (siderophores production, phosphate solubilizing properties and organic acids production), were streaked on Luria-Bertani (LB) agar medium supplemented with glycine. A filter paper soaked in picric acid (0.5%; *w*/*v*), and sodium carbonate (2%; *w*/*v*) was placed in the upper lid of the Petri plate [22]. Plates were incubated at 28 °C for 5 days. Changes in the filter paper colour from yellow to orange, red or brown were considered positive.

PGPR isolates that tested positive for siderophores production, phosphate solubilizing properties, and organic acids production, were identified through molecular methods. These isolates were grown in liquid LB medium, at 30 °C, for 24 h. Genomic DNA was extracted [23], and DNA concentration was determined using a NanoDrop 2000 UV-Vis spectrophotometer (Thermo Fisher Scientific, Wilmington, DE, USA). Bacterial ribosomal subunit 16S gene was amplified using universal primers (*27F*: 5′-AGAGTTTGATCCTGGCTCAG-3′ and *1492R*: 5′-GGTTACCTTGTTACGACTT-3′) [24]. Amplification was performed using DFS-*Taq* DNA Polymerase (Bioron, Römerberg, Germany) and the thermocycling program: 94 °C for 7 min; 30 cycles of 94 °C (30 s), 50 °C (30 s) 72 °C (90 s), and a final extension step at 72 °C for 10 min. Amplification products were sequenced using *1492R* primer, at Macrogen (Amsterdam, The Netherlands). Obtained sequences were blasted against available sequences (NCBI), using the BLAST algorithm. Identification was based on *e*-value, higher similarity identity and on ecological considerations.

### 2.3. PGPR Effects on Arabidopsis Thaliana Root-Architecture

Those bacterial isolates that were previously found to be capable of siderophores production, phosphate solubilisation and organic acids production were grown in liquid LB medium, at 30 °C, for 24 h. PGPR growth was evaluated by densitometry at 600 nm (OD600) and adjusted to 1.0. *Arabidopsis thaliana* (ecotype Columbia; Col-0) seeds were sterilized by submersion on solution of ethanol (70%; *v*/*v*) with Sodium Dodecyl Sulphate (SDS; 0.05%; *w*/*v*) for 3–5 min, followed by immersion in ethanol for 10–20 s (adapted from [25]). Eight seeds were plated on a straight line in Murashige and Skoog (MS) agar medium, 1 cm away from the upper end of plate. Seeds were stratified for 48 h at 4 °C and were then vertically incubated at 21 °C, with a fixed photoperiod of 16 h of light and 8 h of darkness, for 96 h. A bacterial suspension (200 μL; OD600 = 1) was placed in a parallel line, 5 cm away from the seeds. Plates with no bacterial inoculation were used as control. For each PGPR, each treatment and control were performed in quadruplicate (4 plates x 8 seeds). Root morphological parameters, including primary root length, number of lateral roots, and root hairs presence, were evaluated at 3-, 6- and 9-days post inoculation (dpi).

### 2.4. PGPR Antagonistic Activity against B. mediterranea and D. corticola

The same previously selected PGPR isolates, characterized by displaying multiple PGPR traits, were grown in liquid LB medium, at 30 °C, for 24 h. PGPR growth was evaluated by densitometry (OD600) and adjusted to 1.0. Isolates were pre-screened in vitro for antifungal activity against the cork oak phytopathogens, *B. mediterranea* and *D. corticola*. Both endophytic phytopathogens have been previously isolated from cork oak trees showing mild disease symptoms in Grândola region [26]. Fresh PDA plates were divided into four quadrants and a 5 mm agar plug from actively growing mycelium (*D. corticola* or *B. mediterranea*) was placed in the centre. A drop of 2.5 µL of each PGPR suspension was inoculated onto the agar surface, on each quadrant, 1 cm away from the edge of the plate. PDA plates were incubated at 25 °C, in the dark, and results were evaluated after 3 days for *D. corticola* and 5 days for *B. mediterranea* assays. Those PGPR strains that induced a visible mycelial inhibition were further studied through in vitro antagonism assays against both phytopathogenic fungi, using a dual culture method [27]. For this, PGPR isolates were grown for 24 h in LB medium and OD600 was adjusted to 1.0. A drop of 2.5 µL of bacterial suspension was positioned opposed to a fungal plug, and both were placed 2.5 cm away from the edges of the plate. Plates were incubated at 25 °C, in the dark, for 7 days for *D. corticola* and 9 days for *B. mediterranea* assays. Incubation periods were determined according to the requirement for each phytopathogen to reach the plate edge in controls. All antagonism assays were performed in triplicate. Fungal mycelial area was measured at 3-, 5- and 7-days for *D. corticola* and 5-, 7- and 9-days for *B. mediterranea*, using ImageJ software (Copyright 1993, 2016, Oracle). The percentage of growth inhibition (PGI) was calculated using the following formula: PGI = [(AC–AB)/AC] × 100, where AC is the area of pathogenic fungal growth in the control plate, and AB is the area of pathogenic fungus growing in the presence of PGPR.

### 2.5. Data and Statistical Analyses

Differences between isolated bacteria obtained from distinct isolation temperatures and bioclimates were determined by two-way ANOVA and Tukey’s multiple comparison tests, using the Windows GraphPad Prism 6.01 program (GraphPad Software, La Jolla, CAUSE). Evolutionary distances between identified species and phylogenetic tree construction were based on the Maximum Composite Likelihood method [28] and evolutionary analyses were conducted in MEGA X [29]. Comparisons were made using the Neighbor-Joining method [30]. Differences in primary root length and number of lateral roots were determined over time, considering each PGPR, bioclimate and temperature used for bacterial isolation by one-way ANOVA, followed by Dunnett’s multiple comparison tests, using the Windows GraphPad Prism 6.01 program (GraphPad Software, La Jolla, CAUSE). PGI (%) was determined and statistical analysis was performed by one-way ANOVA tests (Dunnett’s multiple comparison tests) using the Windows GraphPad Prism 6.01 program (GraphPad Software, La Jolla, CAUSE).

## 3. Results

### 3.1. Identification of PGPR from Cork Oak Forests Soils

**Figure 1:**
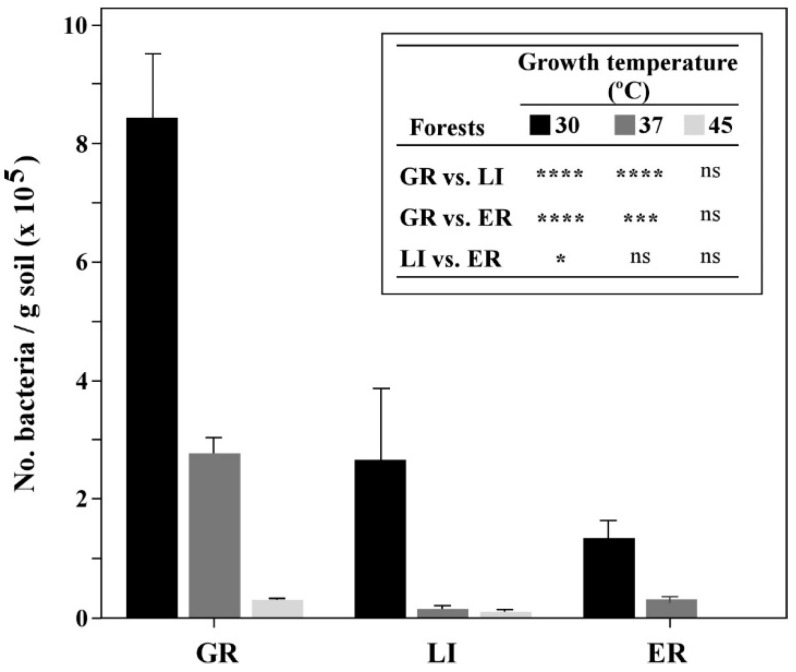
Titer of bacterial communities detected in each forest soil, according to bacterial growth temperatures used. Statistically significant differences are displayed on the side-table and significance levels are represented by * (*p* ≤ 0.05), *** (*p* ≤ 0.001) and **** (*p* ≤ 0.0001); Non-significant differences are displayed with (ns).

A total of 7634 colony-forming units (CFU) were isolated from the cork oak soil samples from GR (3988 CFU; 52.2%), LI (2192 CFU; 28.7%) and ER (1454 CFU; 19%). As expected, the number of CFUs decreased with increasing temperatures used for growing bacteria, being the highest number of CFUs registered at 30 °C and the lowest at 45 °C (Figure 1). Regarding semi-arid forest (GR), 48.7% of all CFUs were detected at 30 °C, but for sub-humid forest (LI) and humid forest (ER), 72.9% and 73.8% were isolated at 30 °C. Semi-arid forest (GR) also presented higher CFU abundance at 30 °C and 37 °C (*p* ≤ 0.001) when compared to sub-humid (LI) and humid forests (ER).

From each forest and isolation growth temperature group, a subsample of bacterial colonies was randomly isolated for further studies. At the end, a total of 324 single colonies were isolated (GR-104; LI-120; ER-100) and used for studying PGPR features (Table S1). From all the tested bacterial isolates, only 16% (51 isolates; 25 from GR, 14 from ER and 12 from LI) presented positive results for all four biochemical tests (Figure S1). After testing HCN production in these 51 isolates, only one isolate (AJ11 *Serratia quinivorans*) from LI forest soil was capable of producing HCN. From the 51 bacterial isolates, only 43 were able to be sequenced, the remaining being hereafter referred to as *Unidentified*. From the sequenced samples, 42 were successfully identified up to at least genera level, sample AJ46 being hereafter named as *Unknown*. Identified bacterial isolates (Table 1) mainly belonged to *Firmicutes* (24 isolates), which was represented exclusively by *Bacillus* genus [*B. megaterium* (18 isolates), *B. cereus* (3), *B. simplex* (1), *B. nakamurai* (1) and another *Bacillus* sp. (1)]. The remaining isolates belonged to *Proteobacteria* (18 isolates), comprising three bacterial families [*Enterobacteriaceae* (11 isolates), *Yersiniaceae* (6) and *Pseudomonadaceae* (1)]. *Enterobacteriaceae* was represented by three genera [*Cedecea* (5), *Klebsiella* (4), and *Ewingella* (2)], *Yersiniaceae* by two genera [*Rouxiella* (4) and *Serratia* (2)], while *Pseudomonadacae* was individually represented by *Pseudomonas mohnii* (1). ER presented a total of six genera identified (*Rouxiella*, *Serratia*, *Klebsiella*, *Ewingella*, *Pseudomonas* and *Bacillus*), followed by LI and GR, with three (*Serratia*, *Cedecea* and *Bacillus*) and two (*Klebsiella* and *Bacillus*) genera, respectively. While *Bacillus* was present in all sampled forests, *Cedecea* (LI), *Serratia* (ER), *Ewingella* (ER) and *Pseudomonas* (ER) were exclusively present in most humid forests. On the other hand, the semi-arid forest was highly enriched in *Bacillus megaterium* (14 out of all 18 identified isolates).

Phylogenetic analysis of 16S gene from identified bacterial species was performed to analyse the pattern of bacterial distribution according to cork oak forests. Phylogenetic tree presented two clades, one with similarities to species from *Bacillus* genus (clade 1) and the other with similarities to species from other genera (clade 2; Figure S2). Twenty-five sequences were considered within the *Bacillus* clade, which was subdivided into three subclades. *B. megaterium* aligned in two different subclades (subclades 1.1 and 1.2), while other *Bacillus* species clustered in a single subclade (subclade 1.3). AJ46 isolate, previously named as *Unknown*, clustered into subclade 1.2, making it a potential member of *Bacillus* genus, closely related to *B. megaterium* species. Phylogenetic evaluation results have also revealed that clustering patterns did not form according to forest geographic location and bioclimates.

### 3.2. Arabidopsis Thaliana Root Modulation by Cork Oak Soil PGPR

Specific effects of each PGPR (51 isolates) on *A. thaliana* primary root architecture was evaluated by a dual culture method. To better understand the dynamics over time, primary root length, number of lateral roots and presence/absence of root hairs were evaluated at 3-, 6- and 9-days after PGPR inoculation. From all tested PGPR, 33.33% displayed a consistent suppressive effect on primary root length (*p* ≤ 0.0001, Table S2). The most notorious effects were detected in isolates of *Bacillus* (5 isolates of *B. megaterium* reducing at least 50% of total primary root length and 1 isolate of *B. nakamurai* reducing almost 35% of total length), *Serratia* sp. (1 isolate reducing almost 50%) and *Cedecea neteri* (3 isolates reducing at least 60%). Some *Unidentified* isolates (4) were also capable of promoting at least 60% root length reduction.

PGPR also modulated lateral root formation in *A. thaliana* (*p* ≤ 0.0001; Table S3). Most PGPR isolates (45) stimulated lateral root formation, but only 6 induced their development at early stage of seedling development (3-dpi). From these, *B. megaterium* (AJ47), *C. neteri* (AJ15), *Rouxiella* sp. (AJ22) and an *Unidentified* isolate (AJ13) induced the lateral root number during the experiment. For not underestimating PGPR with non-significant differences in the early stages of inoculation, but that still promoted lateral root formation by the end of the assay, lateral roots induction (LRI %) was calculated in relation to control and over time. It was possible to understand that besides the previous selected PGPR, other *B. megaterium* (AJ40 and AJ61) isolates were able to highly increase the lateral roots formation. Besides these, *Klebsiella aerogenes* (AJ45) and *P. mohnii*. (AJ25), as well as an *Unknown* (AJ46, a putative *Bacillus* sp.) and *Unidentified* (AJ51 and AJ62) isolates, also highly induced the lateral roots formation.

Compared to the control, where no root hairs ever developed during the entire assay, the promoting effects of PGPR on root hairs incidence became evident over time (Table S4). By 3 dpi, PGPR that induced the highest percentage of seedlings with root hairs development were *B. megaterium* (AJ50 and AJ53) and an *Unidentified* isolate (AJ56). The ability of inducing root hairs increased with incubation time. At the end of the experiment (9 dpi), from the PGPR isolates that promoted root hairs formation in more than 75% of *A. thaliana* seedlings, most were *B. megaterium* (7 out of 8).

Taken together, most PGPR (31 out of 51) induced great outcomes on a single root parameter, but only 3 could simultaneously modulate two root parameters (Table 1). None of them induced root modifications concerning all three evaluated root parameters. Differences between PGPR effects at 9 dpi were compared taking into consideration the temperature used for bacterial isolation. PGPR isolated at 45 °C displayed a more prominent suppressive effect on primary root length than PGPR isolated at 30 °C or 37 °C (*p* ≤ 0.05). On the other hand, lateral root development was not dependent on PGPR isolation temperature (*p* > 0.05). Lastly, analysing the PGPR effects on root hairs formation, a high increase of root hairs presence was more evident in higher isolation temperatures (37 °C or 45 °C), as weaker root hair inducers were isolated at 30 °C. Therefore, out of the three evaluated root-parameters, the development of primary root and root hairs seemed to be the most affected by PGPR isolation methods.

### 3.3. Serratia spp. and Bacillus spp. as Key Genera for Controlling Cork Oak Bark Pathogens

From all 51 PGPR isolates, only 15 presented antifungal activity against *B. mediterranea* and/or *D. corticola* fungal growth inhibition (Table 1). Results revealed that 5 isolates inhibited both phytopathogens growth, 3 isolates presented antifungal activity against *B. mediterranea* and 7 isolates against *D. corticola*. Those PGPR able to inhibit *B. mediterranea* were isolated from all three sampled forests, although they were more prevalent in moister places (5 isolates from humid ER and 2 from semi-humid LI) than semi-arid forest (1 isolate from GR). In contrast, PGPR presenting antifungal activity against *D. corticola* were similarly isolated from semi-arid (5 isolates, GR) and humid forests (5 isolates, ER; 2 isolates, LI). PGPR isolates that presented antifungal activity against both phytopathogens were isolated from moister forests (3 isolates, ER; 2 isolates, LI).

**Figure 2:**
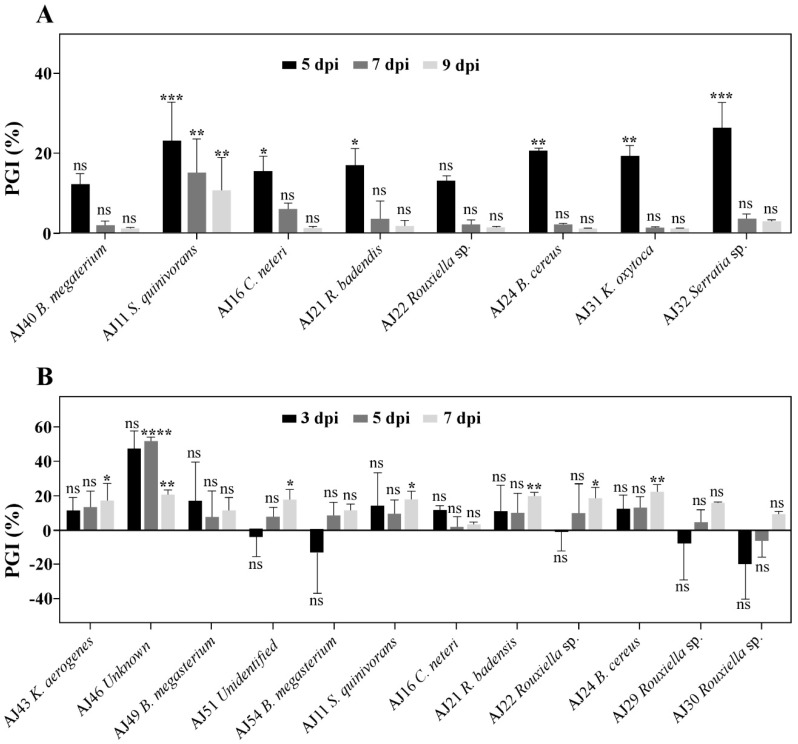
Antifungal activity of selected PGPR against *B. mediterranea* (**A**) and *D. corticola* (**B**). The percentage of fungal growth inhibition (% PGI) was determined at 5-, 7- and 9 dpi for *B. mediterranea* and at 3-, 5- and 7 dpi for *D. corticola*, considering the difference of pathogen growth area in the presence of each PGPR, in relation to the area of unchallenged pathogen (in the absence of PGPR, corresponding to PGI = 0%). Bars represent mean values (*n* = 3) and error bars represent SE. Asterisks represent statistically significant differences in relation to control for each PGPR, at *p* ≤ 0.05 (*), *p* ≤ 0.01 (**), *p* ≤ 0.001 (***) and *p* ≤ 0.0001 (****). Non-significant differences are displayed with (ns).

All antagonistic PGPR isolates were further studied for their antimicrobial activity over time. PGPR isolates presented a reduction of inhibitory activity against *B. mediterranea* with time (Figure 2A). Among tested PGPR, 6 isolates significantly inhibited mycelial growth (*p* ≤ 0.05) at 5 dpi, but from 7 dpi on, only *S. quinivorans* maintained a significant (*p* ≤ 0.01) antagonistic activity against *B. mediterranea*. Also, from all assayed PGPR, *Serratia* genus (*Serratia* sp. and *S. quinivorans*) presented the highest growth inhibition (PGI) at 5 dpi (26.23% and 23.07%; *p* ≤ 0.001, respectively). The antifungal activity of *S. quinivorans* was further studied by following the cultural features over time (Figure S3). As previously detected, the bacterial antagonistic behaviour was significantly higher at early stages of inoculation (*p* ≤ 0.001), where the highest antagonistic activity was displayed 5 days after incubation (Figure S3A). At 7 dpi, an accumulation of a yellowish pigment at the contact front of *B. mediterranea* mycelium became evident (Figure S3B).

The antagonistic effect of PGPR against *D. corticola* were similarly assessed over time (Figure 2B). Due to the increased growth rate of this pathogen in relation with *B. mediterranea*, a higher variation and lack of reproducibility was detected in this assay. In contrast with *B. mediterranea* antagonists, no PGPR revealed significant inhibitory activity by 3 dpi and only the *Unknown* isolate (AJ46) significantly inhibited *D. corticola* mycelial growth (*p* ≤ 0.0001) at 5 dpi. However, at 7 dpi, most assayed PGPR (7 isolates) presented significant antagonistic activity (*p* ≤ 0.05). Among these, *Rouxiella badensis* AJ21, *B. cereus* AJ24 and *Unknown* AJ46 isolates revealed the highest inhibitions (*p* ≤ 0.01). In any case, due to the high variation detected, further investigation is necessary for supporting and complementing these findings. *Unknown* AJ46 and *B. cereus* were further studied regarding their cultural features over time (Figure S4). After 7 dpi, *D. corticola* hyphae began to suffer morphological alterations, such as irregular branching, increased diameter, and irregular growth directions during interactions (Figure S4C,D).

## 4. Discussion

Taking into consideration the creation of a PGPR collection that could provide multifunctional services to cork oak stands, soil bacterial isolates were obtained from cork oak forests in distinct bioclimates and using different temperatures for selecting bacteria. The idea was to get the most diversified isolates, already adapted to different environmental conditions for foreseeing future biocontrol and biofertilizer strategies for plant protection. As expected, the highest number of CFU was detected in 30 °C, as the most soil microorganisms are mesophilic, presenting the maximal growth temperatures between 25 °C and 35 °C [31]. Nevertheless, the most arid Portuguese forest (GR) exhibited an increased proportion of bacterial isolates able to grow at higher temperatures, thus suggesting a more adapted bacterial community to those conditions than the rainiest forests. In accordance, bacteria exposed to higher stress levels, caused by drought and/or high temperatures have been reported to exhibit enhanced adaptation to such stressful environmental pressures. For example, bacteria native from areas with limited water conditions are more qualified to deal with stress, when compared to bacteria from irrigated areas [32]. The resistance to higher temperatures has been described to result from different cellular adaptations, such as the production of heat-stable enzymes, synthesis of heat shock proteins (known for their ability to lessen and protect cellular damage from increased temperatures), or even endospores formation [13,33,34]. From the spore-forming bacteria, *Bacillus* (*Firmicutes*) was the only identified genus in all sampled forests. Among these, *B. megaterium* was the most frequent species identified in semi-arid forest (GR) and also the single one to be identified when using heat-forcing selection. Due to the ability of producing endospores, *Bacillus* species have been reported to exhibit an increased resilience to extreme conditions, such as water deficiency, high temperatures and high levels of UV radiation, having been isolated from arid, semi-arid and desertic climates [33,35].

Bacteria displaying all assayed PGPR traits (siderophore production, phosphate solubilisation, and organic acids production) were also able to change plant root-architecture, namely by reducing primary root growth, or by increasing lateral root or root hairs formation. However, only three isolates were proficient in inducing more than a single root morphologic trait. Furthermore, the most proficient PGPR in promoting a specific root alteration rarely significantly affected other morphological aspect, suggesting specificity and even antagonism among suppressing/promoting abilities of PGPR on root architecture. This behaviour could be related with the inducing factors that are behind root morphological changes. Alterations in root morphology have been related with modulation of auxin levels, namely in indole-3-acetic acid (IAA) concentrations [36]. Indeed, different IAA concentrations are described to result in different root formations (primary or lateral roots, or root hairs; [37]). The combination of IAA produced by plant and bacteria should be optimal to promote plant growth and will determine whether bacteria will stimulate or suppress plant growth [38]. For example, in the particular case of primary root, relatively low levels of IAA are required to induce primary root growth and a supplementary source of bacterial IAA results in shorter main roots (e.g., [39]).

Several bacteria from plant rhizosphere possess the ability to produce IAA, including bacteria from *Bacillus* and *Pseudomonas* genera [40,41]. This agrees with the high number of *Bacillus* spp. with inhibitory effects on primary root growth. Unlike primary root, lateral root development has been described to be stimulated through high IAA levels [42,43]. Therefore, using a reductionist approach, PGPR able to reduce *A. thaliana* primary root length, possibly due to high IAA concentrations, would be expected to significantly promote the formation of lateral roots and/or root hairs. In the present work, this was not the case and even a significant positive correlation was found between primary root length and formation of lateral roots (*r* = 0.9033, *p* ≤ 0.0001). This result suggests that the production of IAA by itself does not fully explain the growth-suppressing/stimulating abilities by PGPR, and other phytohormone levels (e.g., ethylene) and/or factors could also modulate root development and elongation [44]. Most interestingly, such factors seem to be dependent on bacteria background (forest and/or isolation temperature) rather than genotype. Indeed, distinct isolates from the same species (e.g., *B. megaterium*) revealed different suppressing/stimulating root morphogenic features, which were dependent from bacteria background (forest and/or isolation temperature). For example, *B. megaterium* isolated at lower temperatures (30 °C and 37 °C) displayed lower primary root suppression rates, when compared to *B. megatarium* isolated at 45 °C (Table 1 and Supplementary Table S1). Also, from all evaluated root-parameters, the formation of root hairs seemed to be the root trait most affected by the forest bioclimate, since the most proficient *B. megaterium* in inducing root hair formation were found in semi-arid forest (6 out of 7 isolates).

Besides biochemical traits and root phenotypic alterations, PGPR were also evaluated for their antifungal activity against bark cork oak disease agents (*B. mediterranea* and *D. corticola*). Several PGPR presented an in-vitro inhibitory effect against *B. mediterranea* that decreased over time, probably due to the lapse production of antifungal compounds or to the defensive mechanisms created by the fungus. Nevertheless, *S. quinivorans* (AJ11) revealed an inhibitory activity that lasted throughout the full antagonist assay, possibly due to the production of the volatile HCN. Indeed, in this study, only *S. quinivorans* was identified as a HCN producer, a volatile antifungal compound capable of inhibiting growth of several plant pathogens [45]. Other *Serratia* genus members have been also reported as active producers of different lytic enzymes and several antibiotics, as well as HCN producers [46,47]. Inhibition of *B. mediterranea* growth has been described to be affected by microbial volatiles produced by the cork oak beneficial endophytes, *Coniothyrium carteri* and *Fusarium oxysporum* [25]. In contrast with *B. mediterranea*, the antifungal activity of PGRP against *D. corticola* suggested increasing antagonistic effect with time that could be explained by a dose-dependent effect of antimicrobial compound(s). The isolates *Unknown* (AJ46; a close *B. megaterium* isolate) and *B. cereus* (AJ24) resulted in *D. corticola* hyphal modifications, similar to those reported with known synthetic antimicrobial compounds [48]. Due to their late effect, extended inoculation periods could be further explored.

The use of PGPR as biofertilizers has been increasing because they facilitate the overall plant growth and yield of multiple crops in an eco-friendly manner [49]. Although they are well-described for enhancing agricultural yields, few studies on PGPR isolated from non-agricultural fields and their importance for soil sustainability have been reported. The ability of recruiting the most appropriated nutrient metabolizers is essential for plant trees and to the global forest nutrient cycle. In this study, *Bacillus* members, mainly *B. megaterium* recovered from the driest forest, were singled out as displaying increased nutrient solubilization traits (siderophore production, phosphate solubilization, and organic acids production) and by inducing root beneficial morphological changes. Being already adapted to cork oak forests, these isolates could hold a promising tool for enhancing cork oak development and sustainability. Members of *Bacillaceae* were recently described to play an essential role on cork oak forests mycorrhiza establishment.

## 5. Conclusions

This work resulted in the creation of a PGPR collection from cork oak forests that display increased nutrient solubilisation traits (siderophore production, phosphate solubilisation, and organic acids production), induce a root phenotype as described for other plant-beneficial microorganisms, and are able to antagonize cork oak bark phytopathogens. Suppressing/promoting abilities on root architecture were found to be specific from each PGPR and depended on the environment where bacteria reside, and method used for their isolation. PGPR communities, mainly from semi-arid environments and from *Bacillus* genus, could hold a promising tool to enhance plant development, even under stressful environmental circumstances. Such natural occurring plant beneficial microorganisms are in increasing demand due to their role in promoting more sustainable practices, not only in agriculture and crop production, but also in forestry.

## Figures and Tables

**Table 1:** Effects of selected PGPR on *A. thaliana* (*At*) root architecture and their antifungal activity against cork oak pathogens. *Arabidopsis* root development was evaluated considering the primary root growth induction (PRGI), lateral roots induction (LRI) and root hair presence (IRH). Displayed values represent data at 9 dpi and the most noticeable effects are highlighted in grey, as detailed in Tables S2–S4. Antifungal activity against *B. mediterranea* (*Bmed*) and *D. corticola* (*Dcort*) of selected PGPR, detected after five and three days, respectively, is also highlighted in grey. Isolates are discriminated according to their origin (cork oak forest) and temperature used for isolation.

Forest	Temp	Code	Identification	*At* Root Development			Antifungal Activity	
				PRGI (%)	LRI (%)	IRH (%)	*Bmed*	*Dcort*
GR	30 °C	AJ40	*Bacillus megaterium*	95.8	602.1	59.4	+	−
		AJ41	*Bacillus megaterium*	81.3	442.6	50.0	−	−
	37 °C	AJ42	*Bacillus megaterium*	64.1	527.7	75.0	−	−
		AJ43	*Klebsiella aerogenes*	67.7	445.7	71.9	−	+
		AJ44	*Klebsiella aerogenes*	90.1	455.3	93.8	−	−
		AJ45	*Klebsiella aerogenes*	88.5	731.9	43.8	−	−
		AJ46	*Unknown*	106.3	829.8	66.7	−	+
		AJ47	*Bacillus megaterium*	66.2	538.3	37.5	−	−
		AJ48	*Bacillus megaterium*	63.0	394.7	41.7	−	−
		AJ49	*Bacillus megaterium*	71.4	425.5	41.7	−	+
		AJ50	*Bacillus megaterium*	64.0	531.9	96.9	−	−
		AJ51	*Unidentified*	64.6	658.5	40.6	−	+
		AJ52	*Bacillus* sp.	67.7	541.5	68.8	−	−
		AJ53	*Bacillus megaterium*	58.9	386.2	96.9	−	−
	45 °C	AJ54	*Bacillus megaterium*	57.8	523.4	54.2	−	+
		AJ55	*Bacillus megaterium*	67.2	535.1	78.1	−	−
		AJ56	*Unidentified*	64.6	404.3	58.3	−	−
		AJ57	*Bacillus megaterium*	55.2	456.4	87.5	−	−
		AJ58	*Bacillus megaterium*	57.3	552.1	90.6	−	−
		AJ59	*Unidentified*	60.9	525.5	59.4	−	−
		AJ60	*Unidentified*	56.8	519.1	50.0	−	−
		AJ61	*Bacillus megaterium*	88.5	671.3	50.0	−	−
		AJ62	*Unidentified*	72.9	625.5	58.3	−	−
		AJ63	*Unidentified*	56.8	528.7	56.3	−	−
		AJ64	*Bacillus megaterium*	57.3	498.9	68.8	−	−
LI	30 °C	AJ10	*Unidentified*	80.2	439.4	21.9	−	−
		AJ11	*Serratia quinivorans*	64.1	594.7	34.4	+	+
		AJ14	*Cedecea neteri*	59.9	435.1	53.1	−	−
	37 °C	AJ8	*Cedecea* sp.	60.4	425.5	43.8	−	−
		AJ9	*Bacillus megaterium*	71.9	445.7	37.5	−	−
		AJ12	*Cedecea neteri*	56.8	495.7	34.4	−	−
		AJ13	*Unidentified*	58.9	495.7	37.5	−	−
		AJ15	*Cedecea neteri*	63.5	469.1	53.1	−	−
		AJ16	*Cedecea neteri*	59.9	412.8	31.3	+	+
		AJ17	*Bacillus megaterium*	83.9	564.9	94.0	−	−
		AJ18	*Bacillus simplex*	66.7	390.4	33.3	−	−
	45 °C	AJ19	*Bacillus megaterium*	76.6	485.1	43.8	−	−
ER	30 °C	AJ21	*Rouxiella badensis*	76.6	442.6	31.3	+	+
		AJ22	*Rouxiella* sp.	64.6	581.9	46.9	+	+
		AJ23	*Bacillus mycoides*	67.2	519.1	53.1	−	−
		AJ24	*Bacillus cereus*	87.5	588.3	65.6	+	+
		AJ25	*Pseudomonas mohnii*	117.2	788.3	71.9	−	−
		AJ26	*Bacillus cereus*	74.5	392.6	25.0	−	−
		AJ27	*Ewingella americana*	62.5	363.8	41.7	−	−
		AJ28	*Ewingella americana*	73.4	525.5	56.3	−	−
		AJ29	*Rouxiella* sp.	66.2	558.5	29.2	−	+
	37 °C	AJ30	*Rouxiella* sp.	70.8	452.1	45.8	−	+
		AJ31	*Klebsiella oxytoca*	50.0	219.1	0.0	+	−
		AJ32	*Serratia* sp.	52.6	292.6	20.8	+	−
	45 °C	AJ33	*Bacillus megaterium*	49.0	359.6	21.9	−	−
		AJ34	*Bacillus nakamurai*	53.7	475.5	28.1	−	−

## Data Availability

The sequences datasets of the 16S rDNA presented in this study are available in NCBI database with accessions MZ700072 to MZ700099.

## Supplementary Materials

The following are available online at https://www.mdpi.com/article/10.3390/microorganisms9091973/s1, Table S1. PGPR traits of bacterial isolates obtained from the three sampled cork oak forests (GR, LI, and ER) and isolated using different incubation temperatures (30 °C, 37 °C and 45 °C). Positive results are highlighted in dark grey and negative results in light grey. The codes of those isolates, displaying all four PGPR traits and used in subsequent experiments, are presented in a box. Figure S1. Venn diagrams of shared and exclusive PGPR traits (A—organic acids production, B—phosphate solubilisation and C—siderophores production) exhibited by bacterial isolates, obtained from different cork oak forests and using different temperatures for bacterial growing. Figure S2. Phylogenetic tree of identified OTUs. Isolates obtained from GR are highlighted in orange, whereas isolates from LI and ER are highlighted in blue and green, respectively. All these isolates are capable of siderophores production, phosphate solubilisation and organic acids production. The only isolate with HCN production ability is highlighted with a red square. Table S2. PGPR effect on *A. thaliana* primary root length development over time—3 dpi, 6 dpi and 9 dpi. Values for primary root length represent the mean of all replica (cm). Primary root growth induction (PRGI %) represents the percentage of primary root length (cm), when in co-inoculation with PGPR, in relation to control. The effect of each PGPR on primary root length is visualized using a heat map, where the most inhibitory effects are displayed in black and the less inhibitory effects are displayed in white. Bacteria presenting a consistent and significant inhibitory behaviour are depicted in bold, and those with the better outcomes are highlighted in grey. Asterisks represent statistically significant differences to control at *p* ≤ 0.05 (*), *p* ≤ 0.01 (**), *p* ≤ 0.001 (***) and *p* ≤0.0001 (****). Table S3. PGPR effect on *A. thaliana* lateral roots development over time—3 dpi, 6 dpi and 9 dpi. Values for number of lateral roots represent the mean of all replica. Lateral roots induction (LRI %) represents the percentage of seedlings with lateral roots, when in co-inoculation with PGPR, in relation to control. The effect of each PGPR on number of lateral roots is visualized using a heat map, where the most stimulating effects are displayed in black and the less stimulating effects are displayed in white. Bacteria presenting a consistent and significant promoting behaviour are depicted in bold, and those with the better outcomes are highlighted in grey. Asterisks represent statistically significant differences to control at *p* ≤ 0.05 (*), *p* ≤ 0.01 (**), *p* ≤ 0.001 (***) and *p* ≤ 0.0001 (****). Table S4. PGPR effect on *A. thaliana* root hairs presence over time—3 dpi, 6 dpi and 9 dpi—compared to control. Root hairs induction (RHI %) represents the percentage of seedlings that developed root hairs, when in co-inoculation with PGPR. The effect of each PGPR on *A. thaliana* seedlings that developed root hairs is visualized using a heat map, where the most stimulating effects are displayed in black and the less stimulating effects are displayed in white. Bacteria that induced root hairs on more than 75% of *A. thaliana* seedlings by 9 dpi are highlighted in grey.
