# Supplementary material for: Cork Oak Forests Soil Bacteria: Potential for Sustainable Agroforest Production
Source: Microorganisms. 2021 Sep 16;9(9):1973. doi: 10.3390/microorganisms9091973 (PMC8472395; doi:10.3390/microorganisms9091973)
Supplement: Supplementary file 1 [file microorganisms-09-01973-s001.zip › microorganisms-1294799-supplementary.pdf]

Supplementary Material

Table S1. PGPR traits of bacterial isolates obtained from the three sampled cork oak forests (GR, LI, and ER) and isolated using different incubation temperatures (30ºC, 37ºC and 45ºC). Positive results are highlighted in dark grey and negative results in light grey. The codes of those isolates, displaying all four PGPR traits and used in subsequent experiments, are presented in a box.

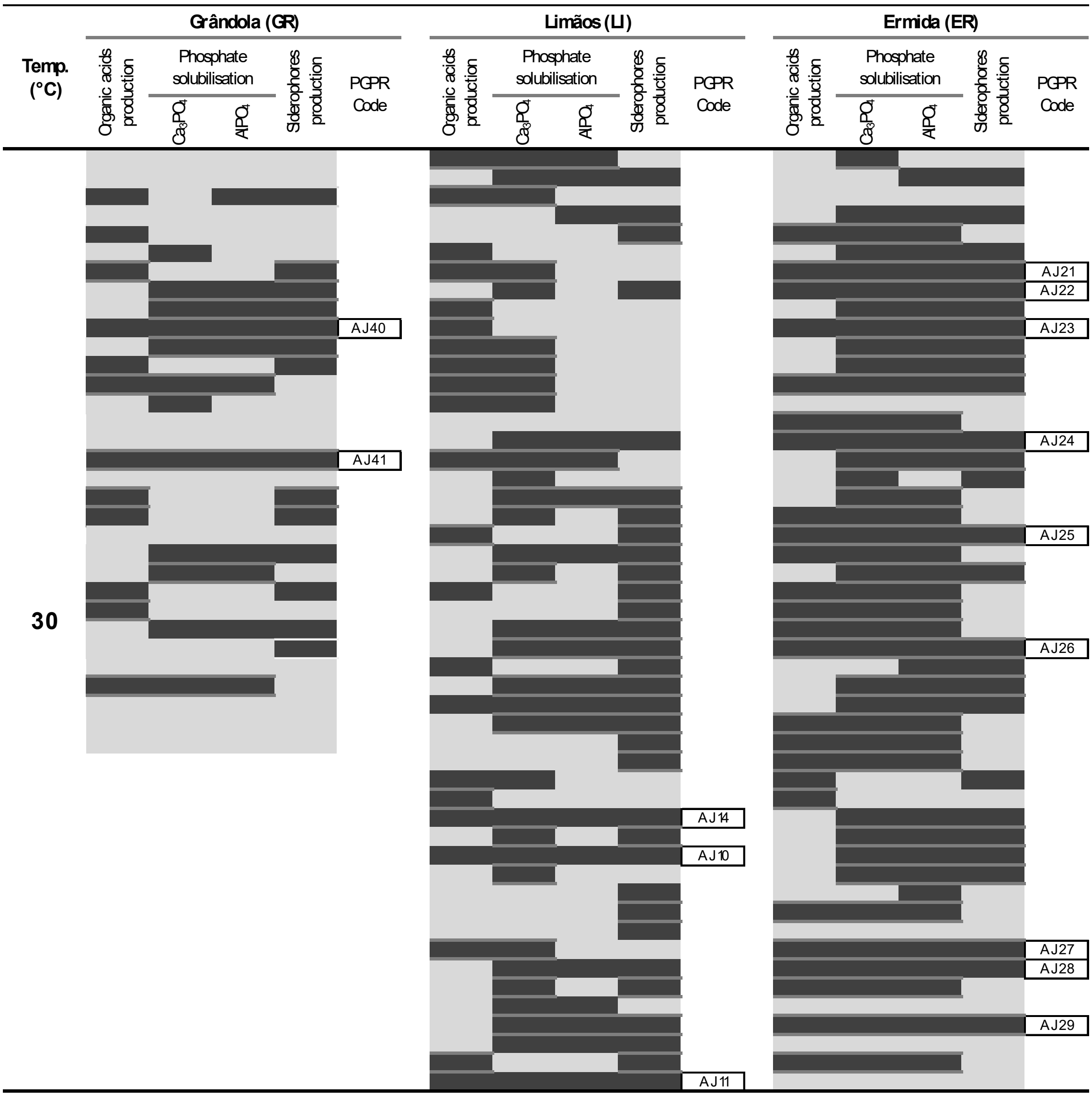

Table S1. *Continuation*

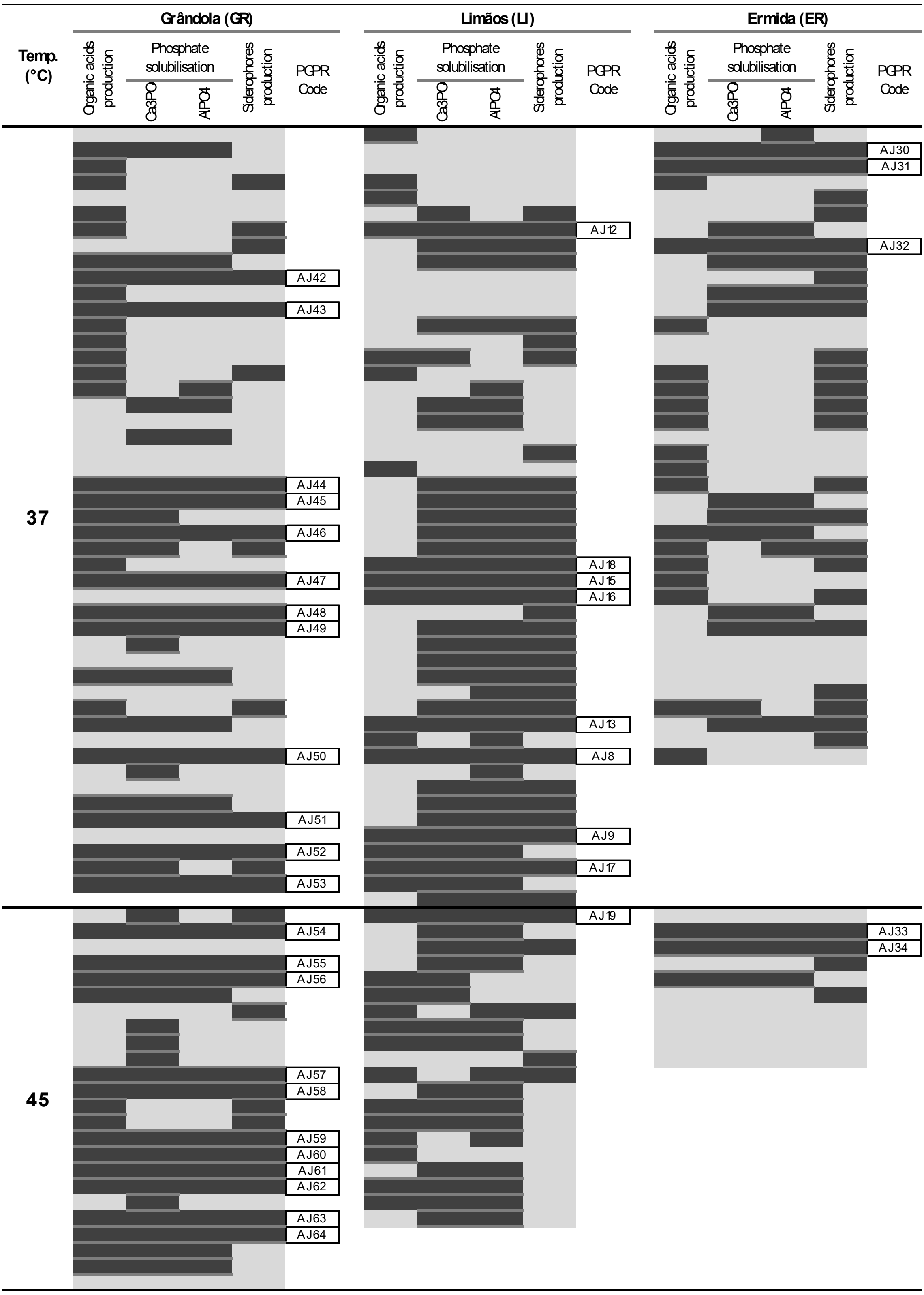

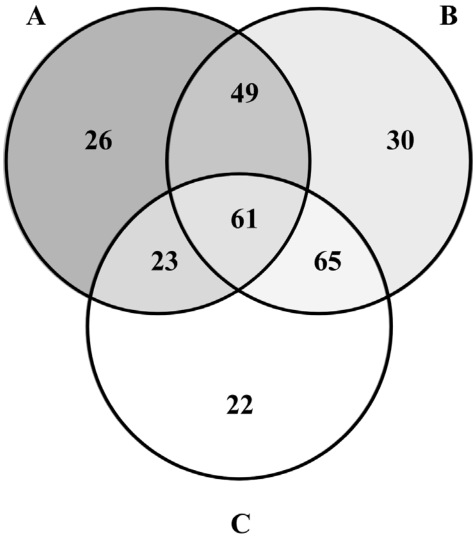

Figure S1. Venn diagrams of shared and exclusive PGPR traits (A - organic acids production, B - phosphate solubilisation and C - siderophores production) exhibited by bacterial isolates, obtained from different cork oak forests and using different temperatures for bacterial growing.

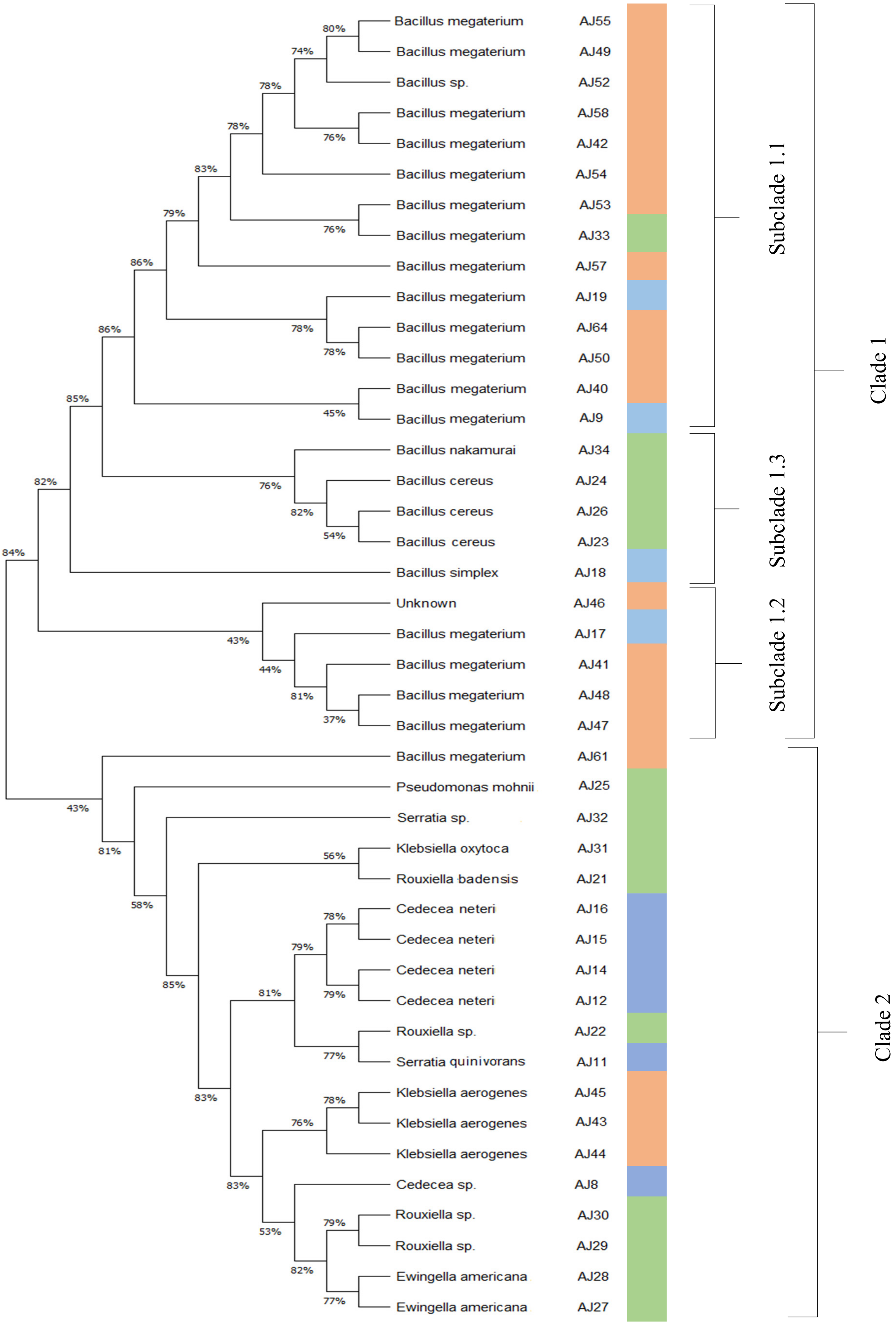

Figure S2. Phylogenetic tree of identified OTUs. Isolates obtained from GR are highlighted in orange, whereas isolates from LI and ER are highlighted in blue and green, respectively. All these isolates are capable of siderophores production, phosphate solubilisation and organic acids production. The only isolate with HCN production ability is highlighted with a red square.

Table S2. PGPR effect on *A. thaliana* primary root length development over time - 3 dpi, 6 dpi and 9 dpi. Values for primary root length represent the mean of all replica (cm). Primary root growth induction (PRGI %) represents the percentage of primary root length (cm), when in co-inoculation with PGPR, in relation to control. The effect of each PGPR on primary root length is visualized using a heat map, where the most inhibitory effects are displayed in black and the less inhibitory effects are displayed in white. Bacteria presenting a consistent and significant inhibitory behaviour are depicted in bold, and those with the better outcomes are highlighted in grey. Asterisks represent statistically significant differences to control at $p \leq 0.05$ (*), $p \leq 0.01$ (**), $p \leq 0.001$ (***) and $p \leq 0.0001$ (****).

Forest	Temp (°C)	Code	Identification	Primary root length (cm)			PRGI (%)			
				3 dpi	6 dpi	9 dpi	3 dpi	6 dpi	9 dpi	Mean
**Control**				1.4	1.5	1.9	100	100	100	100
GR	30	AJ40	*Bacillus megaterium*	1.3	1.4	1.8	92.9	95.3	95.8	94.7
		**AJ41**	***Bacillus megaterium***	**0.9******	**0.8****	**1.6****	61.4	50.7	81.3	64.5
	37	AJ42	*Bacillus megaterium*	0.9**	0.9	1.2	66.4	63.5	64.1	64.7
		AJ43	*Klebsiella aerogenes*	1.0*	1.2	1.3	72.1	77.7	67.7	72.5
		AJ44	*Klebsiella aerogenes*	1.2	1.2	1.7	85.0	81.1	90.1	85.4
		AJ45	*Klebsiella aerogenes*	1.2	1.2	1.7	85.7	83.1	88.5	85.8
		AJ46	*Unknown*	1.5	1.4	2.0	103.6	91.9	106.3	100.6
		AJ47	*Bacillus megaterium*	0.9****	2.0	1.3	65.7	131.8	66.2	87.9
		AJ48	*Bacillus megaterium*	1.0*	0.9	1.2	70.0	59.5	63.0	64.2
		AJ49	*Bacillus megaterium*	1.0**	1.1	1.4	69.3	72.3	71.4	71.0
		**AJ50**	***Bacillus megaterium***	**1.0****	**0.8***	**1.2***	69.3	56.1	64.1	63.1
		**AJ51**	***Unidentified***	**1.0***	**0.8****	**1.2***	74.3	54.7	64.6	64.5
		AJ52	*Bacillus* sp.	1.1	1.1	1.3	75.7	71.6	67.7	71.7
		AJ53	*Bacillus megaterium*	0.9****	0.9	1.1**	61.4	60.8	58.9	60.4
	45	**AJ54**	***Bacillus megaterium***	**0.7******	**0.6******	**1.1***	51.4	37.2	57.8	48.8
		AJ55	*Bacillus megaterium*	1.0****	1.0	1.3	70.0	67.6	67.2	68.3
		AJ56	*Unidentified*	1.0**	1.0	1.2	67.9	68.2	64.6	66.9
		**AJ57**	***Bacillus megaterium***	**0.8******	**0.7*****	**1.1*****	55.0	50.0	55.2	53.4
		**AJ58**	***Bacillus megaterium***	**0.7******	**0.6******	**1.1*****	49.3	37.8	57.3	48.1
		**AJ59**	***Unidentified***	**0.8******	**0.7*****	**1.2****	54.3	50.0	60.9	55.1
		**AJ60**	***Unidentified***	**0.7******	**0.6******	**1.1*****	52.9	39.9	56.8	49.8
		AJ61	*Bacillus megaterium*	1.2	1.3	1.7	82.9	89.9	88.5	87.1
		AJ62	*Unidentified*	1.0*	1.0	1.4	72.1	64.9	72.9	70.0
		**AJ63**	***Unidentified***	**0.7******	**0.7*****	**1.1*****	52.1	46.6	56.8	51.9
		**AJ64**	***Bacillus megaterium***	**0.8******	**0.6******	**1.1*****	56.4	37.8	57.3	50.5
LI	30	AJ10	*Unidentified*	1.1	1.1	1.5	79.3	76.4	80.2	78.6
		**AJ11**	***Serratia quinivorans***	**0.9*****	**0.9***	**1.2***	67.1	60.8	64.1	64.0
		**AJ14**	***Cedecea neteri***	**0.8******	**0.8****	**1.2****	62.1	56.1	59.9	59.4
	37	AJ8	*Cedecea* sp.	1.0	1.1	1.2	69.3	73.7	60.4	67.8
		AJ9	*Bacillus megaterium*	1.1	1.1	1.4	75.0	73.0	71.9	73.3
		**AJ12**	***Cedecea neteri***	**0.8******	**0.8*****	**1.1*****	58.6	53.4	56.8	56.2
		**AJ13**	***Unidentified***	**0.8******	**0.8*****	**1.1****	59.3	52.0	58.9	56.7
		**AJ15**	***Cedecea neteri***	**0.9******	**0.9****	**1.2***	64.3	57.4	63.5	61.8
		**AJ16**	***Cedecea neteri***	**0.8******	**0.8*****	**1.2****	60.0	52.0	59.9	57.3
		AJ17	*Bacillus megaterium*	1.2	1.2	1.6	82.9	79.7	83.9	82.2
		AJ18	*Bacillus simplex*	1.0**	1.0	1.3	69.3	68.2	66.7	68.1
	45	AJ19	*Bacillus megaterium*	1.1	1.1	1.5	75.7	73.7	76.6	75.3
ER	30	AJ21	*Rouxiella badensis*	1.1	1.1	1.5	77.1	74.3	76.6	76.0
		**AJ22**	***Rouxiella* sp.**	**0.9******	**0.9****	**1.2***	65.0	58.1	64.6	62.6
		AJ23	*Bacillus mycoides*	1.0***	0.9	1.3	67.9	63.5	67.2	66.2
		AJ24	*Bacillus cereus*	1.2	1.3	1.7	87.1	84.5	87.5	86.4
		AJ25	*Pseudomonas mohnii*	1.6	1.7	2.3	114.3	112.2	117.2	114.6
		AJ26	*Bacillus cereus*	0.9**	0.9	1.4	65.0	59.5	74.5	66.3
		**AJ27**	***Ewingella americana***	**0.8******	**0.9***	**1.2***	59.3	60.1	62.5	60.6
		AJ28	*Ewingella americana*	1.1	1.2	1.4	79.3	83.1	73.4	78.6
		AJ29	*Rouxiella* sp.	0.9***	0.9	1.3	64.3	60.8	66.2	63.8
	37	AJ30	*Rouxiella* sp.	0.9***	1.0	1.4	65.0	64.2	70.8	66.7
		AJ31	*Klebsiella oxytoca*	0.8****	0.9	1.0**	55.7	58.1	50.0	54.6
		**AJ32**	***Serratia* sp.**	**0.7******	**0.8*****	**1.0*****	49.3	50.7	52.6	50.9
	45	**AJ33**	***Bacillus megaterium***	**0.5******	**0.3******	**0.9******	36.4	22.3	49.0	35.9
		**AJ34**	***Bacillus nakamurai***	**0.5******	**0.5******	**1.0******	40.7	32.4	53.7	42.3

Table S3. PGPR effect on *A. thaliana* lateral roots development over time - 3 dpi, 6 dpi and 9 dpi. Values for number of lateral roots represent the mean of all replica. Lateral roots induction (LRI %) represents the percentage of seedlings with lateral roots, when in co-inoculation with PGPR, in relation to control. The effect of each PGPR on number of lateral roots is visualized using a heat map, where the most stimulating effects are displayed in black and the less stimulating effects are displayed in white. Bacteria presenting a consistent and significant promoting behaviour are depicted in bold, and those with the better outcomes are highlighted in grey. Asterisks represent statistically significant differences to control at $p \leq 0.05$ (*), $p \leq 0.01$ (**), $p \leq 0.001$ (***) and $p \leq 0.0001$ (****).

Forest	Temp (°C)	Code	Identification	Lateral roots (No.)			LRI (%)		
				3 dpi	6 dpi	9 dpi	3 dpi	6 dpi	9 dpi
**Contro0l**				0.01	0.25	0.94	100	100	100
**GR**	30	AJ40	*Bacillus megaterium*	0.22	2.47****	5.66****	2200.00	988.00	602.10
		AJ41	*Bacillus megaterium*	0.34	1.97*	4.16**	3400.00	788.00	442.60
	37	AJ42	*Bacillus megaterium*	0.25	2.33**	4.96****	2500.00	932.00	527.70
		AJ43	*Klebsiella aerogenes*	0.31	2.28***	4.19**	3100.00	912.00	445.70
		AJ44	*Klebsiella aerogenes*	0.13	2.47****	4.28***	1300.00	988.00	455.30
		AJ45	*Klebsiella aerogenes*	0.66	2.97****	6.88****	6600.00	1188.00	731.90
		AJ46	*Unknown*	0.63	3.33****	7.80****	6300.00	1332.00	829.80
		AJ47	***Bacillus megaterium***	**0.84****	**2.47*****	**5.06******	8400.00	988.00	538.30
		AJ48	*Bacillus megaterium*	0.21	1.96*	3.71	2100.00	784.00	394.70
		AJ49	*Bacillus megaterium*	0.4	1.72	4.00*	4000.00	688.00	425.50
		AJ50	*Bacillus megaterium*	0.47	2.16**	5.00****	4700.0	864.00	531.90
		AJ51	*Unidentified*	0.59	2.69****	6.19****	5900.00	1076.00	658.50
		AJ52	*Bacillus* sp.	0.41	2.72****	5.09****	4100.00	1088.00	541.50
		AJ53	*Bacillus megaterium*	0.78*	2.31***	3.63	7800.00	924.00	386.20
	45	AJ54	*Bacillus megaterium*	0.08	2.50***	4.92****	800.00	1000.00	523.40
		AJ55	*Bacillus megaterium*	0.50	2.53****	5.03****	5000.00	1012.00	535.10
		AJ56	*Unidentified*	0.36	1.60	3.80	3600.00	640.00	404.30
		AJ57	*Bacillus megaterium*	0.21	1.92	4.29**	2100.00	768.00	456.40
		AJ58	*Bacillus megaterium*	0.19	2.94****	5.19****	1900.00	1176.00	552.10
		AJ59	*Unidentified*	0.09	2.81****	4.94****	900.00	1124.00	525.50
		AJ60	*Unidentified*	0.03	2.69***	4.88****	300.00	1076.00	519.10
		AJ61	*Bacillus megaterium*	0.09	2.97****	6.31****	900.00	1188.00	671.30
		AJ62	*Unidentified*	0.67	2.63****	5.88****	6700.00	1052.00	625.50
		AJ63	*Unidentified*	0.09	2.53****	4.97****	900.00	1012.00	528.70
		AJ64	*Bacillus megaterium*	0.28	2.66****	4.69****	2800.00	1064.00	498.90
**LI**	30	AJ10	*Unidentified*	0.13	1.69	4.13**	1250.00	676.00	439.40
		AJ11	*Serratia quinivorans*	0.63	2.84****	5.59****	6300.00	1136.00	594.70
		AJ14	*Cedecea neteri*	0.53	1.91*	4.09**	5300.00	764.00	435.10
	37	AJ8	*Cedecea* sp.	0.13	2.69***	4.00	1250.00	1076.00	425.50
		AJ9	*Bacillus megaterium*	0.06	1.25	4.19	600.00	500.00	445.70
		AJ12	*Cedecea neteri*	0.69	2.22***	4.66****	6900.00	888.00	495.70
		AJ13	***Unidentified***	**0.81****	**2.44******	**4.66******	8100.00	976.00	495.70
		AJ15	***Cedecea neteri***	**0.75***	**1.91***	**4.41*****	7500.00	764.00	469.10
		AJ16	*Cedecea neteri*	0.41	1.72	3.88*	4100.00	688.00	412.80
		AJ17	*Bacillus megaterium*	0.34	2.97****	5.31****	3400.00	1188.00	564.90
		AJ18	*Bacillus simplex*	0.13	1.42	3.67	1300.00	568.00	390.40
	45	AJ19	*Bacillus megaterium*	0.31	2.25*	4.56*	3100.00	900.00	485.10
**ER**	30	AJ21	*Rouxiella badensis*	0.09	1.53	4.16**	900.00	612.00	442.60
		AJ22	***Rouxiella* sp.**	**0.75***	**2.75******	**5.47******	7500.00	1100.00	581.90
		AJ23	*Bacillus mycoides*	0.44	2.34***	4.88****	4400.00	936.00	519.10
		AJ24	*Bacillus cereus*	0.28	3.25****	5.53****	2800.00	1300.00	588.30
		AJ25	*Pseudomonas mohnii*	0.50	2.88****	7.41****	5000.00	1152.00	788.30
		AJ26	*Bacillus cereus*	0.44	2.31*	3.69	4400.00	924.00	392.60
		AJ27	*Ewingella americana*	0.38	2.38***	3.42	3800.00	952.00	363.80
		AJ28	*Ewingella americana*	0.19	2.13	4.94**	1900.00	852.00	525.50
		AJ29	*Rouxiella* sp.	0.67	2.54****	5.25****	6700.00	1016.00	558.50
	37	AJ30	*Rouxiella* sp.	0.38	2.21**	4.25**	3800.00	884.00	452.10
		AJ31	*Klebsiella oxytoca*	0.50	1.25	2.06	5000.00	500.00	219.10
		AJ32	*Serratia* sp.	1.0***	2.04*	2.75	10000.00	816.00	292.60
	45	AJ33	*Bacillus megaterium*	0.00	0.25	3.38	0.00	100.00	359.60
		AJ34	*Bacillus nakamurai*	0.00	2.44****	4.47***	0.00	976.00	475.50

Table S4. PGPR effect on *A. thaliana'*s root hairs presence over time - 3 dpi, 6 dpi and 9 dpi - compared to control. Root hairs induction (RHI %) represents the percentage of *A. thaliana* seedlings that developed root hairs, when in co-inoculation with PGPR. The effect of each PGPR on *A. thaliana* seedlings that developed root hairs is visualized using a heat map, where the most stimulating effects are displayed in black and the less stimulating effects are displayed in white. Bacteria that induced root hairs on more than 75% of *A. thaliana* seedlings by 9 dpi are highlighted in grey.

Forest	Temp (°C)	Code	Identification	LRI (%)		
				3 dpi	6 dpi	9 dpi
**Control**				0	0	0
GR	30	AJ40	*Bacillus megaterium*	0.0	46.9	59.4
		AJ41	*Bacillus megaterium*	0.0	25.0	50.0
	37	AJ42	*Bacillus megaterium*	0.0	66.7	75.0
		AJ43	*Klebsiella aerogenes*	0.0	65.6	71.9
		AJ44	*Klebsiella aerogenes*	0.0	93.8	93.8
		AJ45	*Klebsiella aerogenes*	3.1	28.1	43.8
		AJ46	*Unknown*	4.2	45.8	66.7
		AJ47	*Bacillus megaterium*	12.5	34.4	37.5
		AJ48	*Bacillus megaterium*	0.0	41.7	41.7
		AJ49	*Bacillus megaterium*	0.0	37.5	41.7
		AJ50	*Bacillus megaterium*	50.0	87.5	96.9
		AJ51	*Unidentified*	3.1	28.1	40.6
		AJ52	*Bacillus* sp.	12.5	68.8	68.8
		AJ53	*Bacillus megaterium*	15.6	84.4	96.9
	45	AJ54	*Bacillus megaterium*	12.5	37.5	54.2
		AJ55	*Bacillus megaterium*	6.3	71.9	78.1
		AJ56	*Unidentified*	16.7	25.0	58.3
		AJ57	*Bacillus megaterium*	0.0	62.5	87.5
		AJ58	*Bacillus megaterium*	12.5	75.0	90.6
		AJ59	*Unidentified*	3.1	43.8	59.4
		AJ60	*Unidentified*	6.3	40.6	50.0
		AJ61	*Bacillus megaterium*	0.0	37.5	50.0
		AJ62	*Unidentified*	4.2	37.5	58.3
		AJ63	*Unidentified*	0.0	40.6	56.3
		AJ64	*Bacillus megaterium*	3.1	53.1	68.8
LI	30	AJ10	*Unidentified*	0.0	18.8	21.9
		AJ11	*Serratia quinivorans*	3.1	31.3	34.4
		AJ14	*Cedecea neteri*	6.3	25.0	53.1
	37	AJ8	*Cedecea* sp.	0.0	43.8	43.8
		AJ9	*Bacillus megaterium*	0.0	25.0	37.5
		AJ12	*Cedecea neteri*	12.5	31.3	34.4
		AJ13	*Unidentified*	3.1	21.9	37.5
		AJ15	*Cedecea neteri*	9.4	34.4	53.1
		AJ16	*Cedecea neteri*	0.0	9.4	31.3
		AJ17	*Bacillus megaterium*	0.0	93.8	94.0
		AJ18	*Bacillus simplex*	0.0	29.2	33.3
	45	AJ19	*Bacillus megaterium*	0.0	43.8	43.8
ER	30	AJ21	*Rouxiella badensis*	0.0	28.1	31.3
		AJ22	*Rouxiella* sp.	3.1	43.8	46.9
		AJ23	*Bacillus mycoides*	0.0	46.9	53.1
		AJ24	*Bacillus cereus*	0.0	65.6	65.6
		AJ25	*Pseudomonas mohnii*	9.4	40.6	71.9
		AJ26	*Bacillus cereus*	0.0	12.5	25.0
		AJ27	*Ewingella americana*	0.0	16.7	41.7
		AJ28	*Ewingella americana*	0.0	0.0	56.3
		AJ29	*Rouxiella* sp.	0.0	8.3	29.2
	37	AJ30	*Rouxiella* sp.	0.0	12.5	45.8
		AJ31	*Klebsiella oxytoca*	0.0	0.0	0.0
		AJ32	*Serratia* sp.	0.0	8.3	20.8
	45	AJ33	*Bacillus megaterium*	0.0	0.0	21.9
		AJ34	*Bacillus nakamurai*	6.3	21.9	28.1

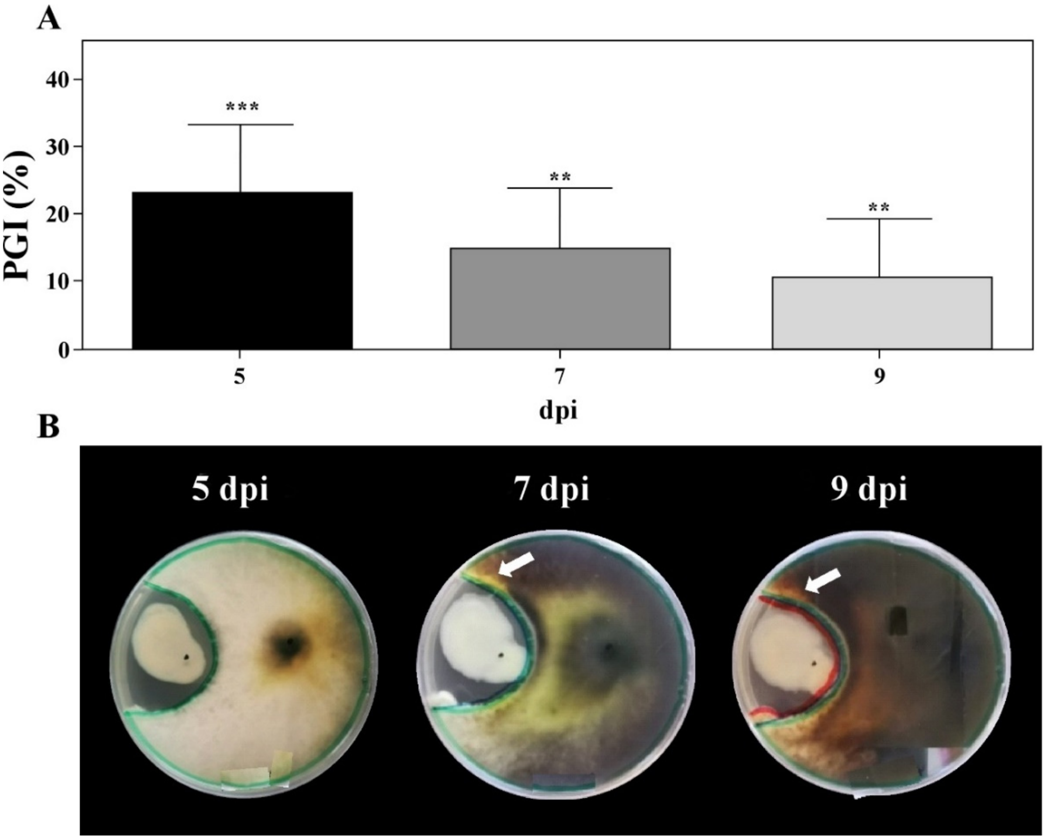

Figure S3. Inhibition of *B. mediterranea* growth by *S. quinivorans* over time (A) and corresponding cultural features (B). Asterisks represent statistically significant differences of fungal growth in relation to control at $p \leq 0.01$ (**), $p \leq 0.001$ (***). White arrows point to a region where the pigment develops.

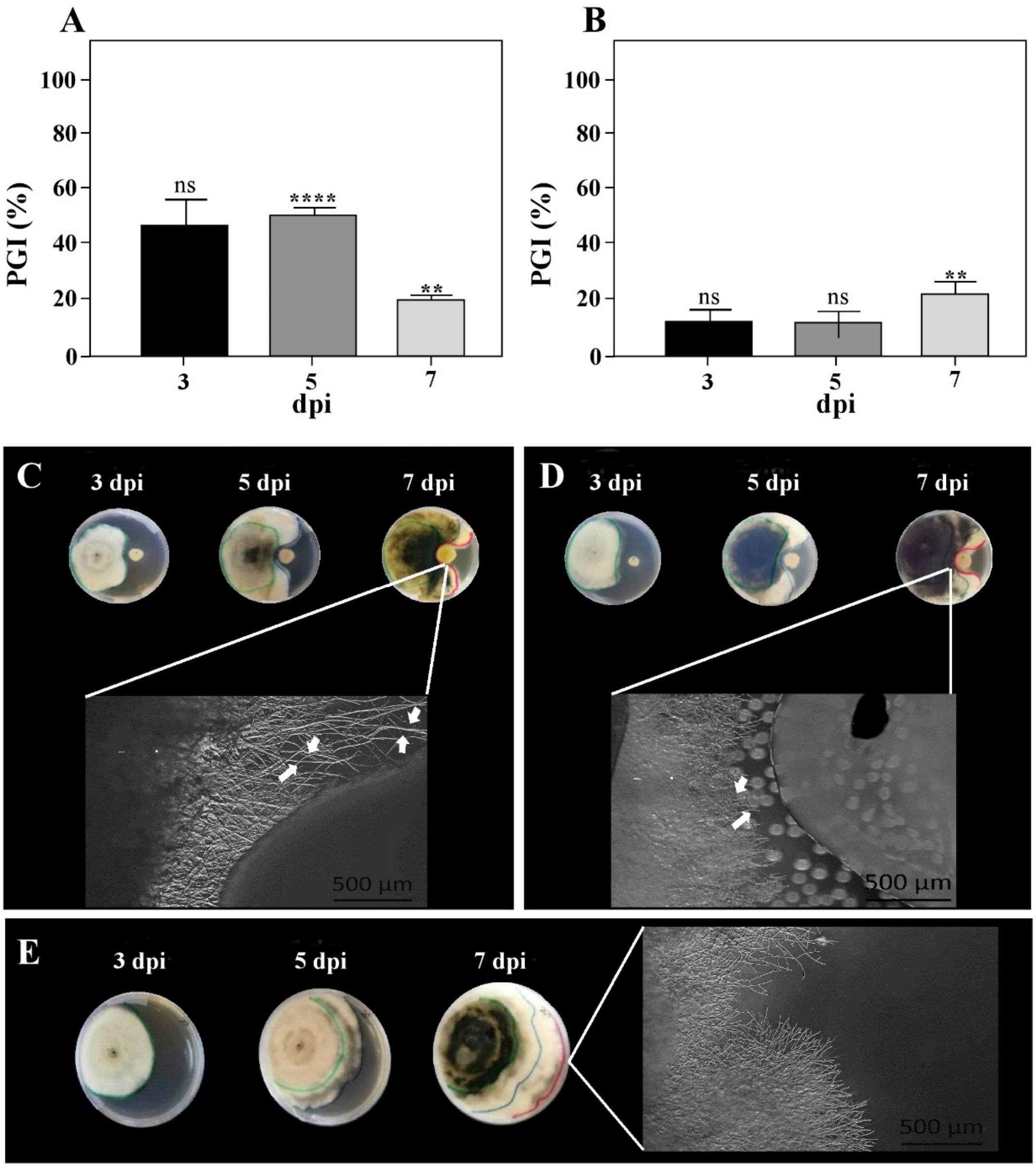

Figure S4. Inhibition of *D. corticola* growth by *Unknown* AJ46 (A) and *B. cereus* AJ24 (B) over time and corresponding cultural features (C and D, respectively). Asterisks represent statistically significant differences to control at $p \leq 0.01$ (**) and $p \leq 0.0001$ (****). Non-significant differences are displayed with (ns). *D. corticola* hyphal modifications in the interaction region comprised an increased diameter and irregular growth directions (white arrows). For comparison, cultural features from control (E), where no PGPR inoculation occurred, are shown.
